# The PDK1–Rsk Signaling Pathway Controls Langerhans Cell Proliferation and Patterning

**DOI:** 10.4049/jimmunol.1501520

**Published:** 2015-09-23

**Authors:** Rossana Zaru, Stephen P. Matthews, Alexander J. Edgar, Alan R. Prescott, Diego Gomez-Nicola, André Hanauer, Colin Watts

**Affiliations:** * Division of Cell Signaling and Immunology, College of Life Science, University of Dundee, Dundee DD1 5EH, United Kingdom;; † Centre for Biological Sciences, University of Southampton, Southampton General Hospital, Southampton SO16 6YD, United Kingdom; and; ‡ Institut de Génétique et de Biologie Moléculaire et Cellulaire, Centre National de la Recherche Scientifique Unite Mixté de Recherche 7104, INSERM U 964, University of Strasbourg, 67404 Illkirch, France

## Abstract

Langerhans cells (LC), the dendritic cells of the epidermis, are distributed in a distinctive regularly spaced array. In the mouse, the LC array is established in the first few days of life from proliferating local precursors, but the regulating signaling pathways are not fully understood. We found that mice lacking the kinase phosphoinositide-dependent kinase 1 selectively lack LC. Deletion of the phosphoinositide-dependent kinase 1 target kinases, ribosomal S6 kinase 1 (Rsk1) and Rsk2, produced a striking perturbation in the LC network: LC density was reduced 2-fold, but LC size was increased by the same magnitude. Reduced LC numbers in Rsk1/2^−/−^ mice was not due to accelerated emigration from the skin but rather to reduced proliferation at least in adults. Rsk1/2 were required for normal LC patterning in neonates, but not when LC were ablated in adults and replaced by bone marrow–derived cells. Increased LC size was an intrinsic response to reduced LC numbers, reversible on LC emigration, and could be observed in wild type epidermis where LC size also correlated inversely with LC density. Our results identify a key signaling pathway needed to establish a normal LC network and suggest that LC might maintain epidermal surveillance by increasing their “footprint” when their numbers are limited.

## Introduction

Immune surveillance in the skin is mediated by different dendritic cell (DC) populations including Langerhans cells (LC), which form a network in the epidermis (1, 2). They differ from the other DC populations in that they originate from the yolk sac and the fetal liver and colonize the epidermis just before birth (3, 4). During the first week of life they differentiate into LC and undergo a rapid but transient burst in proliferation that establishes their unique network. In the adult, at the steady-state, the homeostasis of the LC network is maintained through a balance of in situ proliferation of local precursors and a slow but constant migration to the skin draining lymph nodes (2, 5–8). Only upon skin injury, such as in the case of UV irradiation, are LC replaced first by monocytes, which transiently repopulate the epidermis, and then by bone marrow precursors, which provide long-term reconstitution (5, 9).

The mechanisms behind LC development have been the subject of intense recent investigation. TGF-β1 produced by both keratinocytes and LC has been shown to be essential to maintain the LC network and prevents their spontaneous migration to lymph nodes (10–12). IL-34 produced by keratinocytes is also required for the development of LC (13, 14). Two transcriptions factors, Id2 and runx3, acting downstream of TGF-β1 have been reported to be essential for LC differentiation in the steady-state (15–17). More recently, a role for the kinase complex mTORC1 and p14, a subunit of the LAMTOR complex, that can regulate mTOR function have been identified as important for the proliferation and survival of LC (18, 19), in the case of p14, by maintaining functional TGF-β1 signaling (20). Thus, although some of the factors governing the development and maintenance of LC have been identified, our understanding of the required intracellular signaling pathways is at an early stage.

The p90 kDa ribosomal S6 kinases (Rsks) are Ser/Thr kinases of the AGC kinase family that require activation by both phosphoinositide-dependent kinase 1 (PDK1) and Erk1/2 in most cell types (21). In DCs, Rsks can also be activated by MK2/3 acting downstream of p38 MAPK (22, 23). PDK1 is required for the development of T cells (24) and B cells (25) but is dispensable for the development of macrophages and granulocytes (25, 26). Downstream of PDK1, Rsks regulate several cellular processes including cell proliferation, survival, transcription, translation, and metabolism (21). Because IL-34 is known to activate Erk1/2 (27), one of the upstream activators of Rsk, we assessed the contribution of Rsks to LC biology taking advantage of newly generated mice lacking multiple Rsk isoforms. In this article, we show that the PDK1–Rsk pathway is critical for establishment and maintenance of a normal LC network in mice. In addition, we present evidence that LC may respond to reduced numbers with an increase in LC surface area or “footprint.”

## Materials and Methods

### Mice

Rsk1 and Rsk2 single-knockout (KO) mice were generated as described by Yang et al. (28) and Laugel-Haushalter et al. (29) and maintained on a Bl6/C57 background. Rsk1 and Rsk2 double-KO mice were generated by crossing Rsk1 and Rsk2 single-KO mice. Hematopoietic deletion of PDK1 was achieved by crossing floxed PDK1 mice with Vav-Cre transgenic mice as described by Venigalla et al. (25). Mice were used between 6 wk and 6 mo of age and were bred and maintained under specific pathogen-free conditions. Animal experimentation was approved by the University of Dundee Animal Ethics Committee and was done under a U.K. Home Office Project License.

### Immunofluorescence and cell-size measurement

Ear splits from adult mice or skin from the trunk of 1- or 5-d-old mice were incubated in 0.5M ammonium thiocyanate for 20 min at 37°C. Epidermis was separated from the dermis, fixed for 25 min in 4% paraformaldehyde in PBS, blocked with 1% BSA in PBS, and permeabilized with 0.3% Triton X-100 in PBS for 5 min. Epidermal sheets were stained with Alexa 488–labeled class II MHC Abs (M115/4 clone; Biolegend), FITC-labeled CD45 Abs (eBioscience), or biotin-labeled Langerin Abs (eBioscience) followed by Alexa 555–labeled streptavidin (Invitrogen) and DAPI (Molecular Probes). For each mouse, 5–10 fields were chosen at random and 0.9-μm thick sections were collected on a LSM700 confocal microscope (Zeiss) with a 40× objective. Maximum intensity projections were made and class II MHC^+^ cells/mm^2^ were counted. Cell-surface area and volume were measured using Volocity software (Perkin Elmer). Automatic settings were used to find objects (Otsu’s method) in each image. The offset was set at −65. Some images were analyzed using SD of intensity >2.3. Objects <200 or >5000 μm^3^ were excluded. Objects were filtered by long axis <100 μm. Surface area is defined as the total area of triangles fitted to the surface voxels of the data set. Each image was checked manually to remove cell doublets and hair.

### In vitro LC migration

Ear splits were floated on RPMI 1640 medium supplemented with 10% FCS, non-essential amino acids, Na pyruvate, kanamycin and beta-mercaptoethanol (epidermis facing down) for 0 (control) or 72 h at 37°C. The epidermis was removed, fixed, and stained with anti–MHC class II Abs as described earlier. Five to 10 fields were chosen, and 0.9-μm-thick sections were collected on a LSM700 confocal microscope (Zeiss) with a 40× objective, and number of LC/field was quantified.

### Flow cytometry

Ear splits were incubated in 2.4 U/ml Dispase II (Roche) for 1 h 30 min at 37°C. The epidermis was separated from the dermis and both tissues were incubated in 2% FCS-RPMI 1640 with 2 mg/ml collagenase D and 100 μg/ml DNAse I (Roche) for 45 min at 37°C. Skin draining lymph nodes or spleen was digested in 2% FCS-RPMI 1640 with 2 mg/ml collagenase D and 100 μg/ml DNAse I for 30 min at 37°C. Epidermis, dermis, and lymph node cell suspensions were stained on ice for 30 min with A488-labeled anti-CD45, PE-labeled anti-CD40, PerCP-Cy5.5–labeled anti-CD86, allophycocyanin-labeled anti-EpCAM, e700-labeled anti-CD11c, PE-labeled CD103 or FITC, and e700-labeled anti–MHC class II (eBioscience) Abs. Spleen cell suspension was stained with allophycocyanin-labeled anti-CD11c (BD Biosciences), FITC-labeled anti-Siglec H, PerCP-Cy5.5–labeled anti-CD11b and e700-labeled anti-CD8a Abs (eBioscience). Bone marrow cell suspension was stained FITC-labeled anti-Siglec H and allophycocyanin-labeled anti-B220 Abs (eBioscience). The analysis was performed on a BD Fortessa (BD biosciences). For intracellular staining, cells were fixed with 2% paraformaldehyde/PBS for 10 min at 37°C, permeabilized with 90% MeOH for 20 min on ice, and stained with A488-labeled anti-CD45, PE-labeled anti–MHC class II, and anti–phospho-S6 Abs (Cell Signaling).

### Cell-cycle analysis

Epidermal single-cell suspensions were incubated with 15 μg/ml Hoechst 33342 (Molecular Probes) in 2% FCS in PBS for 30 min at 37°C. Cells were stained with A488-labeled CD45 and PE-labeled MHC class II Abs (eBioscience) on ice. TOPRO-3 (Molecular Probes) was used as a live/dead exclusion marker. Fluorescence was analyzed by flow cytometry as described earlier.

### Bone marrow chimera and UV irradiation

Five- to 6-wk-old littermate or age-matched CD45.1 mice were irradiated 3 h apart with 12 Gy in total. One hour after the last irradiation, mice were injected i.v. with 10^6^ wild type (WT) or Rsk1/2-null bone marrow cells. Three to 4 wk after irradiation, one ear was exposed to UV light as in Merad et al. (5). Three to four weeks later, epidermis was removed, and LC density and size were assessed as described earlier.

### Brain immunohistochemistry

Brain sections were stained and analyzed as described by Gómez-Nicola et al. (30, 31).

### Statistics

Unpaired *t* test and two-way ANOVA were used for the statistical analysis of the data (Prism 6, GraphPad Software); *p = 0.01–0.05, **p = 0.001–0.01, and ***p < 0.001.

## Results

### PDK1 is required for LC development in the epidermis

**FIGURE 1.**
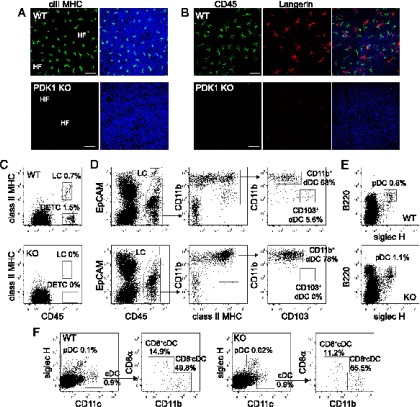
LC are absent in PDK1^fl/fl^ Vav Cre^+ve^ mice. (A and B) Immunofluorescence of PDK1^+/+^ Vav Cre^+ve^ (WT) (A and B) or PDK1^fl/fl^ Vav Cre^+ve^ (PDK1 KO) (**A** and **B**) epidermal sheets stained with DAPI (blue), MHC class II (green) (A), CD45 (green) (B), or Langerin (red) Abs (B). Scale bar, 50 μm. (**C**–**F**) Flow-cytometry analysis of DC populations in epidermis (C), dermis (D), bone marrow (E), and spleen (F) of PDK1^+/+^ Vav Cre^+ve^ (WT) control mice and PDK1^fl/fl^ Vav Cre^+ve^ (KO) mice. Percentage for each DC population is indicated, as well as the gating strategy (arrows). Data are representative of two experiments each done with three PDK1^fl/fl^ Vav Cre^+ve^ mice and three PDK1^+/+^ Vav Cre^+ve^ control mice. cDC, conventional DC; dDC, dermal DC; HF, hair follicles; pDC, plasmacytoid DC; rDC, resident DC.

To investigate the role of PDK1 in LC homeostasis, we crossed PDK1^fl/fl^ mice with Vav-Cre^+ve^ mice to delete PDK1 in the hematopoietic compartment (25). Epidermal sheets were stained with class II MHC Abs, a marker of LC. Remarkably, the epidermis from PDK1^fl/fl^/Vav-Cre^+ve^ mice completely lacked LC (Fig. 1A). This was not simply due to a loss of class II MHC expression because no LC were detected using other markers such as CD45, Langerin, CD11c, and high levels of EpCAM (Fig. 1B and data not shown). Moreover, when we assessed the presence of LC by flow cytometry using the hematopoietic marker CD45, we found that dendritic epidermal T cells (DETC) were also absent (Fig. 1C), supporting previous data showing that PDK1 is essential for T cell development (24, 32). The absence of LC was not the result of an enhanced spontaneous migration to the dermis because no CD45^+^EpCAM^+^ class II MHC^+^ LC were found in that compartment (Fig. 1D). Whereas resident dermal CD103^+^ DCs were absent in PDK1^fl/fl^/Vav-Cre^+ve^ mice, dermal CD11b^+^ DCs were present (Fig. 1D), indicating that the loss of PDK1 differentially affected the generation of dermal DCs. As shown previously by Venigalla et al. (25), the distribution of DC subsets in the bone marrow, the spleen, and the lymph nodes of PDK1^fl/fl^/Vav-Cre^+ve^ mice was similar to WT mice (Fig. 1E, 1F, and data not shown). Thus, PDK1 is required for the development of LC and CD103^+^ dermal DCs but is dispensable for DC development in lymphoid organs.

### Rsk1 and Rsk2 regulate LC size and density

**FIGURE 2.**
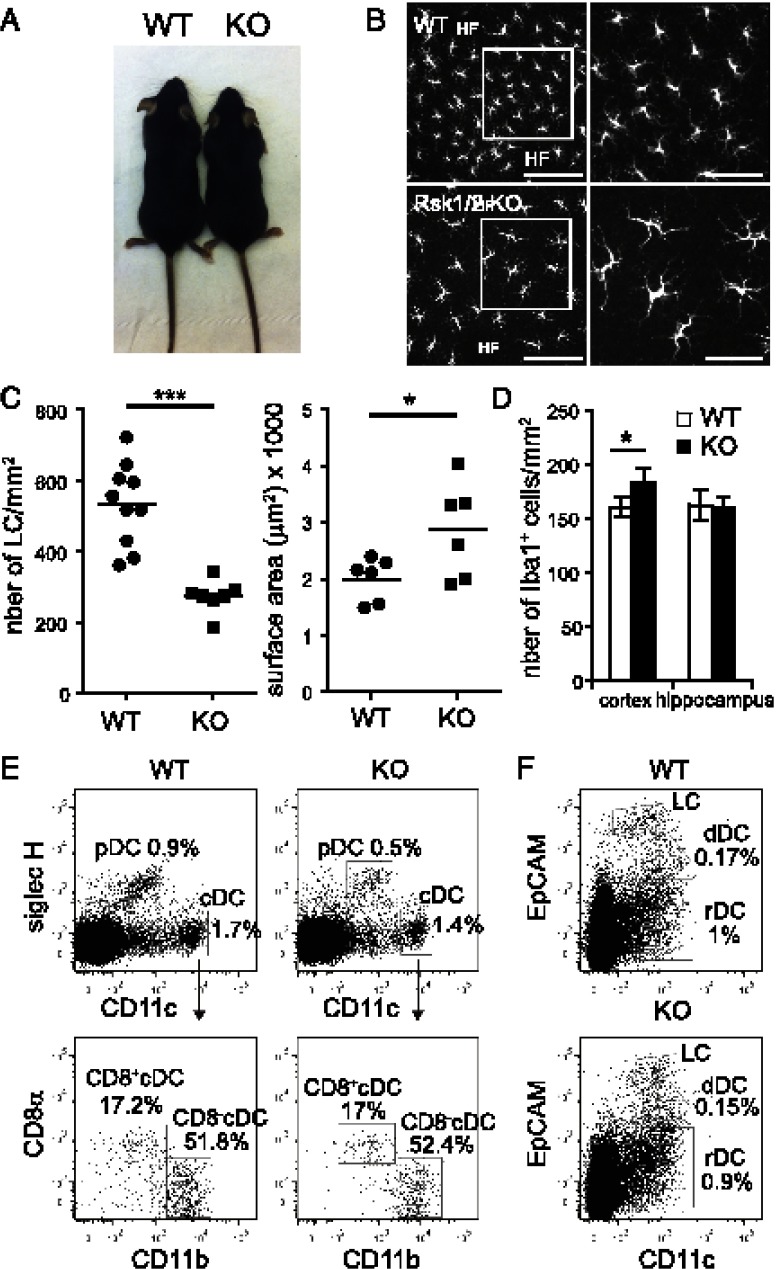
LC are reduced but larger in Rsk1/2-null mice. (**A**) Size of Rsk1/2-null mice (KO) compared with littermate WT control mice. (**B**) Immunofluorescence of WT or Rsk1/2-null epidermal sheets stained with DAPI (blue) and class II MHC Abs (green) to detect LC. Scale bars, 100 μm (*left panels*); 50 μm (*right panels*). *Right panels* represent a magnification of the indicated region in the respective *left panels*. Data are representative of seven independent experiments. (**C**) Density (*left*) and surface area (*right*) of LC (class II MHC^+^ cells) in WT or RSk1/2-null epidermal sheets (density: WT, *n* = 10 mice, KO, *n* = 7 mice; area: WT, *n* = 6 mice, KO, *n* = 6 mice). Individual mice are shown with the mean. (**D**) Density of Iba-1^+^ cells in the cortex and in the hippocampus (*n* = 3 mice/group, 4 fields/mouse). Data are expressed as mean of three biological replicates ± SEM. (**E** and **F**) DC subset distribution in Rsk1/2-null mice. (E) Flow-cytometry analysis of DC populations in spleen (E) and skin draining lymph nodes (F) of Rsk1/2-null or wt control mice. Gating strategy is indicated by arrows. Data are representative of six wt and six Rsk1/2-null mice. cDC, conventional DC; dDC, dermal DC; HF, hair follicles; pDC, plasmacytoid DC; rDC, resident DC. *p = 0.01–0.05, ***p < 0.001.

The earlier results prompted us to investigate which PDK1 downstream effectors were required for LC development. PDK1 activates several downstream Ser/Thr kinases of the AGC family including Rsk, PKB, and S6K (33). Because Rsk isoforms control several biological processes including gene transcription, proliferation, and survival, we asked whether loss of Rsk could impair LC development downstream of PDK1. This was challenging because four Rsk isoforms exist, of which three (Rsk1, Rsk2, and Rsk3) are expressed in LC according to the Immunological Genome Project database (http://www.immgen.org/index_content.html) (34). Single Rsk isoform-deficient mice were viable and had no obvious morphological defects except for Rsk2^−/−^ mice, which, as previously reported, were smaller (29). Moreover, LC were present in normal numbers in mice lacking a single Rsk isoform. Mice lacking both Rsk1 and Rsk2 (hereafter Rsk1/2-null mice) were viable and fertile, but the litters were small and few doubly deficient mice were obtained. Similar to Rsk2^−/−^ mice, their size was reduced compared with littermate controls (Fig. 2A).

When the epidermis of Rsk1/2-null mice was examined, we found that LC were present with normal dendritic morphology and homogenous distribution (Fig. 2B). However, conversely to the epidermis of Rsk1^−/−^ or Rsk2^−/−^ mice, their density was half that of WT (Fig. 2C, Supplemental Fig. 1A). Remarkably, although fewer, LC were substantially increased in size in Rsk1/2-null mice (Fig. 2B, 2C). To investigate whether deleting Rsk3 would have an additional effect, we obtained rare mice lacking Rsk1, Rsk2, and Rsk3. They were viable and had a similar LC phenotype as Rsk1/2-null mice (data not shown), indicating that the regulation of the size and density of LC is mainly regulated by Rsk1 and Rsk2. Rsk1/2-null mice were used for all further experiments.

Rsk1/2 deletion selectively affected LC because other DC subsets including plasmacytoid DCs, CD8^+^ DCs, and CD8^−^ DCs were present in similar numbers in the spleen and lymph nodes of Rsk1/2-null mice and WT controls (Fig. 2E, 2F). Moreover, microglia, which like LC are also long lived and self-renew in situ by proliferation (35, 36), were present in the hippocampus in normal numbers and had a similar size in WT and Rsk1/2-null mice (Fig. 2D and data not shown). There was a modest but significant increase in the number of iba1^+^ microglial cells in the cortex of the Rsk1/2-null mice (Fig. 2D). Taken together, these results reveal a selective requirement for Rsk1/2 for normal LC development.

### Rsk1 and Rsk2 control LC proliferation in newborn and adult mice

**FIGURE 3.**
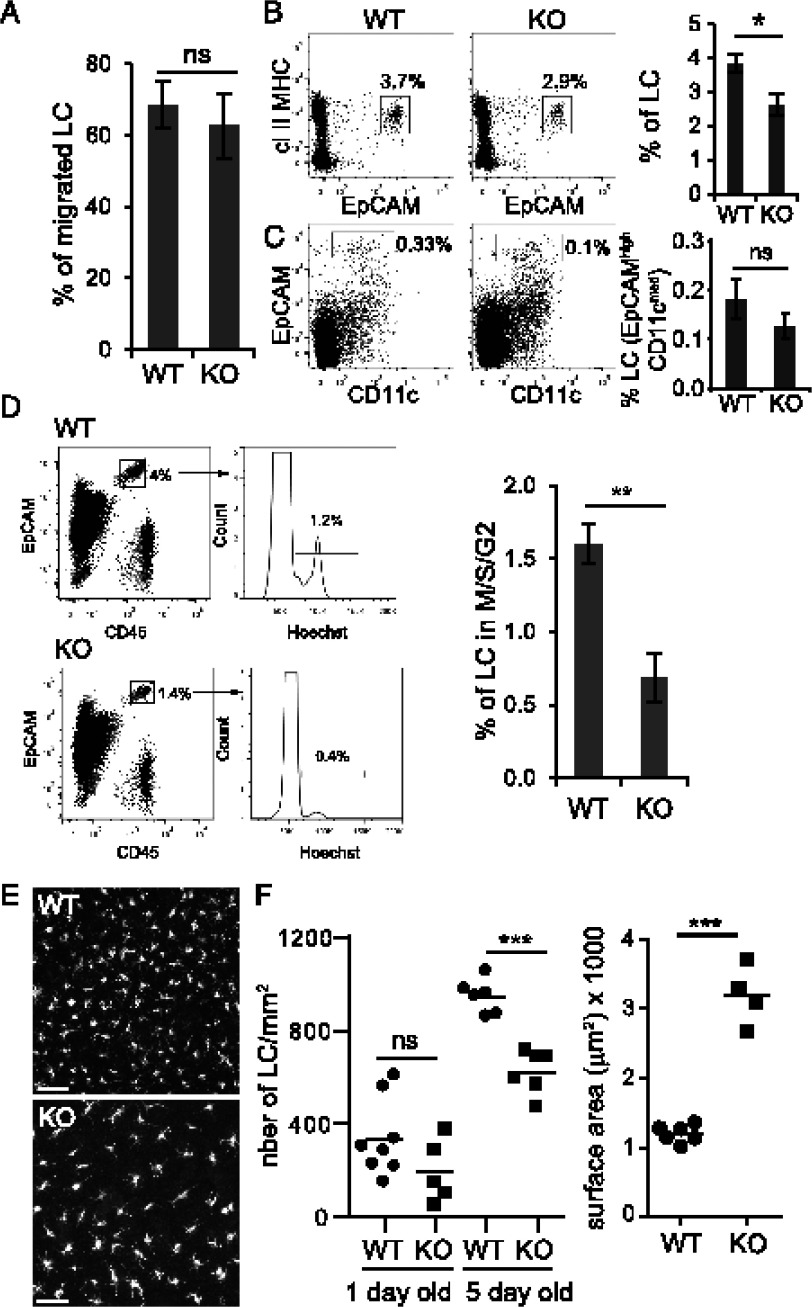
Loss of epidermal LC is not due to enhanced migration but to a proliferation defect. (**A**) WT or Rsk1/2-null (KO) ear splits were incubated for 0 (control) or 72 h at 37°C in RPMI 1640 medium supplemented with 10% FCS, non-essential amino acids, Na pyruvate, kanamycin and beta-mercaptoethanol. Epidermal sheets were isolated, fixed, and stained with MHC class II Abs and analyzed by immunofluorescence to measure LC density. Data are expressed as percentage of LC that have migrated. Data represent the mean of three biological replicates ± SEM and are representative of two experiments. (B and C) Flow-cytometry analysis of CD45^+^ LC in the dermis (**B**) and in the skin draining lymph nodes (**C**) of WT or KO mice. Dot plots (*left*) represent one individual mouse. Histograms show the percentage of LC in the dermis [(B) class II MHC^+^ EpCAM^+^] and in skin draining lymph nodes [(C ), DAPI^−^ CD11c^int^ EpCAM^+^)]. Data represent the mean of four (B) or nine (C) biological replicates ± SEM. (**D**) Cell-cycle analysis of LC (CD45^+^ EpCAM^+^) in the epidermis of WT or KO mice. The percentage of LC in M/S/G_2_ is shown. Data represent the mean of five biological replicates ± SEM. (**E**) Immunofluorescence of epidermal sheets of 5-d-old WT and KO newborn mice stained with MHC class II Abs. Scale bar, 50 μm. Data are representative of six WT and six KO mice. (**F**) Quantification of the number (*left*) of LC/mm^2^ (MHC class II^+^ cells) in 1- or 5-d-old WT or KO epidermal sheets (1 d old: WT = 8 mice, KO = 5 mice; 5 d old: WT = 6 mice, KO = 6 mice) and surface area (*right*) of 5-d-old WT or KO LC (WT = 6 mice, KO = 4 mice). Individual mice are shown with the mean. *p = 0.01–0.05, **p = 0.001–0.01, ***p < 0.001.

Next, we investigated the cause of the reduced LC density. LC homeostasis is maintained by in situ proliferation and by migration to the skin draining lymph nodes (2, 5, 7). We asked whether the migration rate of LC was accelerated in Rsk1/2-null mice, which might explain their reduced number. To measure LC migration, we incubated ear splits in medium for 0 (control) or 3 d and quantified by microscopy LC remaining in the epidermis. As shown in Fig. 3A, the absence of Rsk1/2 did not affect LC migration rate. Fewer LC were found in the dermis of Rsk1/2-null mice (Fig. 3B) and apparently lymph nodes (Fig. 3C), although in the latter case this did not reach significance, most likely because of low and variable LC numbers. Nonetheless, lower numbers in dermis and draining nodes were expected given the reduced numbers in the epidermis.

Because the reduced LC density was not due to an increase in migration, we next investigated whether their capacity to proliferate was affected. We measured the percentage of LC that were cycling by flow cytometry. In WT mice, between 1 and 2% of LC were in M/S/G_2_ phases of the cell cycle (Fig. 3D, Supplemental Fig. 1C), broadly consistent with earlier studies (2, 3). In contrast, only 0.5% of LC were cycling in Rsk1/2-null mice, likely accounting for reduced LC density. The murine LC network is established during the first week of life by a transient burst of LC proliferation (3, 4). As shown in Fig. 3E and 3F, on day 5 after birth the density of LC was again substantially reduced in the epidermis of Rsk1/2-null mice compared with WT mice, showing that Rsk is needed for normal LC development early in adult life. Numbers of LC and their cell cycle status were more difficult to assess reliably in 1-d-old mice because of low numbers of available Rsk1/2-null mice, some uncertainty as to their exact age because cages were not continuously monitored to establish time of birth, and few LC at this stage. Although LC density was apparently reduced in 1-d-old Rsk1/2-null mice, this did not reach significance (Fig. 3F). Similar to the adult epidermis, the shortfall in LC numbers seen in newborn epidermis was accompanied by a striking 3-fold increase in LC size (Fig. 3F).

### Re-establishment of LC in adult mice after inflammation does not require Rsk

**FIGURE 4.**
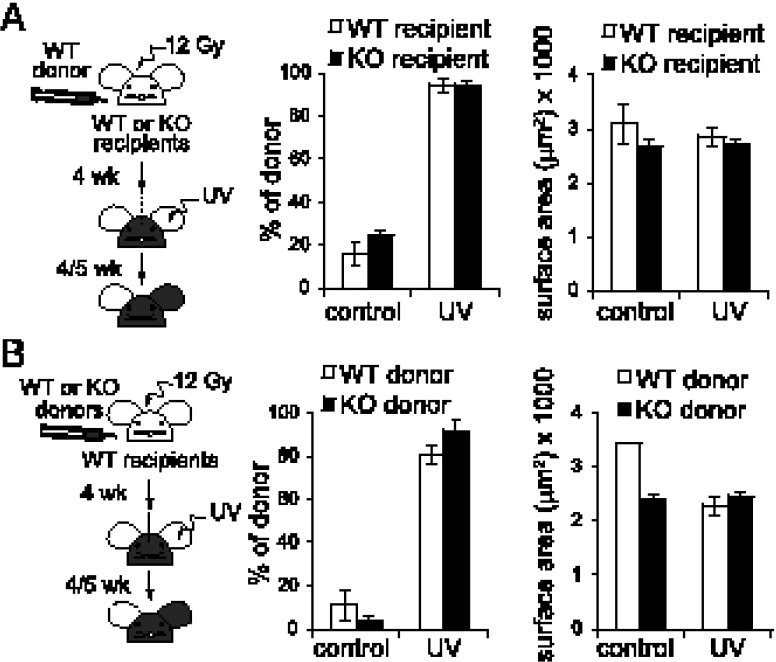
LC require Rsk1/2 for neonatal but not adult repopulation. (**A**) Irradiated WT or KO recipients (CD45.2) were reconstituted with WT bone marrow cells (CD45.1) and UV treated. Epidermal sheets were stained with MHC class II and CD45.2 Abs. Percentage of WT donor-derived LC (class II MHC^+^ CD45.2^−^) with or without UV treatment (*left*) and their surface area (*right*) are shown. (**B**) Irradiated WT recipients (CD45.2) were reconstituted with either WT or KO bone marrow cells (CD45.2) and UV treated. Epidermal sheets were stained with MHC class II and CD45.2 Abs. Percentage of WT or KO donor-derived LC (MHC class II^+^ CD45.2^+^) with or without UV treatment (*left*) and their surface area (*right*) are shown. (A and B) Data are expressed as mean of three biological replicates ± SEM.

We next asked whether the LC phenotype is cell intrinsic or might result from the absence of Rsk1/2 in other cell types present in the epidermis such as DETC and keratinocytes (37). Also, we wanted to establish whether Rsk1/2 were also required when the LC network is ablated and then re-established in the adult from bone marrow precursors. We generated radiation bone marrow chimeric mice where the recipient background was either WT or lacked Rsk1/2. UV-induced inflammation of one ear was used to induce the replacement of recipient LC by donor LC (5). Rsk1/2-null mice reconstituted with WT bone marrow repopulated hematopoietic compartments normally (Supplemental Fig. 2). Five weeks after UV treatment, donor-derived LC were quantified and their surface area measured. As shown in Fig. 4A, the lack of Rsk1/2 in keratinocytes and DETC did not alter the size and the number of repopulating LC relative to WT recipients.

We also made the reciprocal chimera where irradiated WT recipients were reconstituted with WT or Rsk1/2-null donor cells. Reconstitution of T cell, B cell, and DC in the spleen was normal whether donor cells expressed Rsk1/2 (Supplemental Fig. 3), and UV irradiation resulted in efficient loss of host CD45.1 LC and replacement by donor CD45.2 LC (Supplemental Fig. 3E). Moreover, this re-established LC network had similar density and cell-surface area parameters irrespective of whether WT or Rsk1/2-null donor cells were used (Fig. 4B). Thus, an apparently normal LC network can be re-established when Rsk1/2 is absent either in the recipient background or in donor precursors that reconstitute LC. This indicates that Rsk1/2 are required for normal neonatal LC development, but not for re-establishment of the LC network after an inflammatory insult. This may reflect the distinct precursor cell types, extracellular cues, and signaling pathways operating under these different conditions.

### LC size increases in Rsk1/2-null mice are reversible and correlate inversely with LC number

**FIGURE 5.**
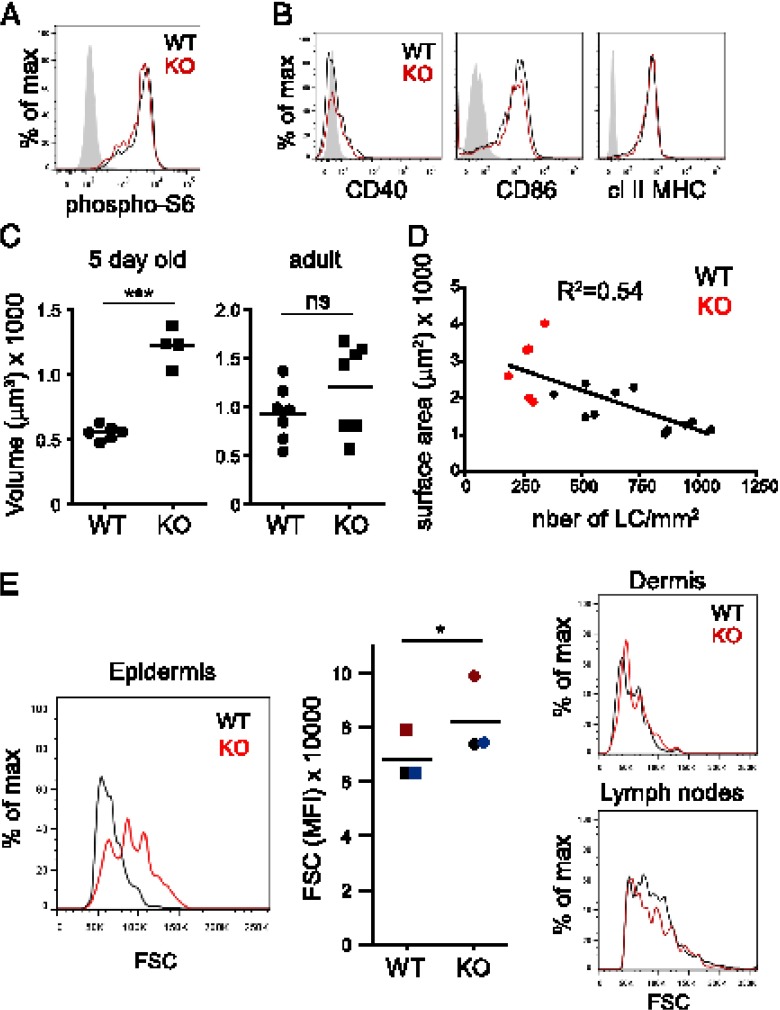
LC may increase their size to maximize epidermal surveillance. (**A**) Flow-cytometry analysis of S6 phosphorylation and CD40, CD86, and class II MHC surface expression (**B**) in WT or KO LC isolated from the epidermis (A and B). Histograms represent one individual mouse for each genotype and are representative of three mice each. (**C**) Measurement of LC volume of WT or KO 5-d-old mice (WT = 6 mice, KO = 4 mice) (*left panel*) and of adult mice (WT = 8 mice, KO = 7 mice) (*right panel*). Individual mice are shown with the mean. (**D**) LC density versus surface area is shown for 11 WT mice (*n* = 11) and KO (*n* = 6) mice. The Pearson coefficient is shown. (**E**) Histograms show the forward scatter analysis by flow cytometry of LC isolated from the epidermis (CD45^+^, class II MHC^+^), dermis (CD45^+^, CD11c^+^, class II MHC^+^, EpCAM^+^), and the skin draining lymph nodes (CD11c^+^, class II MHC^+^, EpCAM^+^) of WT or KO mice. Histograms represent one individual mouse for each genotype and are representative of at least three mice each. *Middle panel* shows the FSC for three independent experiments. Each point is the mean of two mice/genotype/experiment. *p = 0.01–0.05, ***p < 0.001.

Rsk1/2-null LC were not only fewer but also had increased surface area or “footprint” compared with WT LC (Figs. 2C, 3F). We considered several explanations for this striking phenotype. First, increased cell size could be the result of a delay in cell-cycle progression. In other words, Rsk1/2-null LC could be spending more time in the growth phases G_1_ and/or G_2_. Although this might be the case in neonates where LC are proliferating, it is unlikely to account for the size difference of LC in adult mice where only a low percentage of cells are cycling. Second, because a recent report showed that mTORC1 is important in regulating the size and numbers of LC (18), we measured the activity of the mTORC1 activated kinase S6K (33) in WT or Rsk1/2-null LC by flow cytometry. However, LC had similar levels of S6 phosphorylation in the presence or in the absence of Rsk1/2, suggesting that mTORC1 activity is not affected (Fig. 5A). Third, LC activation is known to be accompanied by an increase in cell size (8), raising the possibility that Rsk1/2-null LC might be larger because they have a more activated phenotype. However, epidermal Rsk1/2-null and WT LC expressed similar levels of MHC class II and of the costimulatory molecules CD40 and CD86 (Fig. 5B), indicating that increased LC size is not due to increased maturation of LC in the absence of Rsk1/2. Finally, we considered the possibility that the increase in LC footprint might not be directly linked to the absence of Rsk signaling, but rather was a response triggered by the shortfall in LC numbers.

To explore this further, we measured LC surface area and volume in neonatal and adult epidermis from Rsk1/2-null and WT mice. Interestingly, although 5-d-old Rsk1/2-null mice had a greater volume as well as greater surface area, in adult Rsk1/2-null mice, increased surface LC area was not accompanied by a statistically significant increase in cell volume (Fig. 5C). This implied that in adult mice, overall differences in cell size had normalized but Rsk1/2-null LC had undergone a change in cell shape, which increased their “footprint.” Also, comparing 5-d-old and adult WT mice, we noted that LC density was lower in the latter, but surface area was greater, suggestive of a link between these parameters (Figs. 2C, 3F). We performed two additional experiments to test the notion of an intrinsic inverse relationship between LC density and LC surface area or “footprint.” First, using random sampling of areas of epidermis in multiple WT mice, we measured LC density and LC surface area. As shown in Fig. 5D, LC density varied over a 2- to 3-fold range from mouse to mouse, but importantly so did LC surface area or “footprint,” which correlated inversely. As expected, data points from the Rsk1/2-null mice had the highest LC surface area/LC density area ratio (Fig. 5D). Second, we reasoned that if increased LC size in Rsk1/2-null mice was a tissue-based response to reduced cell density, this should normalize after emigration of LC to dermis and then lymph nodes. Indeed, the greater side-scatter measured by flow cytometry for Rsk1/2-null LC isolated from epidermis normalized when the same cells were sampled in dermis and lymph nodes after emigration from the epidermis (Fig. 5E). Taken together, these results indicate that LC can increase their surface area when LC density is reduced and that the Rsk1/2-null mice illustrate a particularly striking example of this response. Upon exit from the epidermis, this apparently compensatory response is normalized.

## Discussion

Although some of the key cytokines and specific transcription factors needed for LC development and homeostasis have recently been revealed (17), the intracellular signaling pathways that might link the two have only just begun to be investigated. Our study demonstrates for the first time, to our knowledge, a role for two members of the AGC kinase family, namely, PDK1 and its downstream substrates Rsk1 and Rsk2, in the development and maintenance of the LC network. Because no LC were observed in PDK1^fl/fl^/Vav-Cre^+ve^ mice, we reasoned that one or more of its several downstream effector kinases might be critical for LC development. Indeed, mice lacking Rsk1 and Rsk2 had half as many LC in the adult epidermis most likely because of a defect in LC proliferation. Remarkably, LC size was strikingly increased in Rsk1/2-null mice. If Rsk1/2 are activated downstream of PDK1, why do PDK1^fl/fl^/Vav-Cre^+ve^ mice lack all LC but Rsk1/2 mice have only reduced numbers? PDK1 is a constitutively active kinase that regulates >20 downstream target kinases by either PI3K-dependent or -independent mechanisms (33). Thus, partial depletion of LC in Rsk1/2-null mice versus complete lack of LC in PDK1^fl/fl^/Vav-Cre^+ve^ mice suggests that additional PDK1 target kinases besides Rsks are also important for LC development. Indeed, another PDK1 target kinase PKB/Akt is implicated in DC/LC development in mice and humans because of its key role in the PI3K/Akt/mTOR signaling pathway (18, 38).

Despite having been implicated in several biological processes, the study of the Rsk kinases has been hindered by the ubiquitous expression of at least three of the four isoforms (21). The generation of single, double, and triple Rsk1, Rsk2, and Rsk3 KO mice is starting to provide new insights into their roles (29). Lack of Rsk1, Rsk2, or Rsk3 individually had little impact on LC development, but ablation of both Rsk1 and Rsk2 selectively impaired the establishment of LC, but not other DC subtypes. In Rsk1/2-null epidermis, the fraction of LC in cell cycle was only one third of that in WT mice, indicating that reduced LC numbers in adult mice were due to a proliferation defect. Five-day-old Rsk1/2-null mice also had significantly fewer LC, but we could not unequivocally establish that this was due to a defect in the postnatal proliferative burst. For technical reasons, LC numbers and cell cycle status were more difficult to determine accurately on day 1 after birth. Numbers appeared lower in Rsk1/2-null mice, but statistical significance was not obtained. We cannot rule out the possibility that reduced seeding of the epidermis with relevant LC precursors might also occur in Rsk1/2-null mice. Although Rsk1/2 signaling was required for establishment and maintenance of the LC network, it appeared to be dispensable when the network was ablated after an inflammatory insult and restored from bone marrow–derived cells. This may reflect the distinct precursor cell types, extracellular cues, and signaling pathways operating under these different conditions.

Two recent studies have also reported that deficiencies in signaling pathways linked to cell growth and division perturb the LC network (18, 19). Deletion in LC of Raptor, a key subunit of the mTORC1 complex, or p14 (LAMTOR2), an adaptor required for mTORC1 and Erk1/2 signaling from the endosomal/lysosomal compartment, resulted in progressive loss of LC in newborn mice. This was primarily due to enhanced emigration from the skin in the case of Raptor deficiency (18) and reduced LC proliferation in neonates in the case of p14 deficiency (19). In the latter case, reduced levels of TGF-β receptor II render DC/LC hyporesponsive to TGF-β1 (20), which is critical to maintain LC. Rsks are directly linked to these signaling pathways being both downstream effectors of Erk1/2 signaling and upstream activators of mTORC1 signaling (21, 39). Indeed, Rsks can activate mTORC1 by direct phosphorylation of Raptor, as well as by phosphorylation and inactivation of the mTORC1 inhibitor TSC2. The LC phenotype of Rsk1/2-null mice was, however, distinct to those with deficiencies in mTOR signaling. We found no evidence for increased LC apoptosis in Rsk-deficient mice and neither did Rsk-deficient LC migrate faster to draining lymph nodes. Unlike p14 and Raptor-deficient LC, Rsk1/2-null adult mice were able to maintain LC but at a strikingly reduced density.

Rsks have been linked to the promotion of cell proliferation (21), but to our knowledge, only one other study, in Rsk2-deficient T cells, demonstrates that cell-cycle progression in vivo is slowed in the absence of Rsk2 (40). Consistent with a positive role for Rsk in promoting cell cycling, fewer LC were in cycle in Rsk1/2-null mice. Rsks regulate cell-cycle progression through various substrates such as p27kip1 (41, 42) and myt1/cdc2 (43). Analysis of Rsk effectors in LC presents challenges because of their relatively low abundance and the length of time required to isolate them. Nonetheless, we could not detect altered myt1/cdc2 phosphorylation and p27kip levels in Rsk1/2-null LC. Another Rsk substrate is c-fos, which induces cyclin D transcription (44). Indeed, c-fos levels were reduced in Rsk1/2-null LC, but cyclin D levels were not obviously affected (data not shown). Identification of Rsk substrates in LC and other DC will be important, as will the investigation of links between LC growth factor receptors and Rsk. As a potent activator of Erk1/2 signaling (27), IL-34 activates Rsk (data not shown), but whether Rsk is a critical effector of IL-34 signaling remains to be established. Levels of IL-34 and TGF-β mRNA were actually somewhat increased in the epidermis of Rsk1/2-mull mice (data not shown), apparently ruling out deficiency of these key LC-promoting cytokines as an explanation for reduced LC numbers.

LC in Rsk1/2-null mice were not only fewer in number but strikingly increased in size. Rsk1/2-null mice LC had a “footprint” 3- or 2-fold greater than that of their WT counterparts in 5-d-old and adult mice, respectively. The possibility that this was simply due to cell growth without cell division is ruled out, at least in adults, because only 2–3% of LC are proliferating. Nor did Rsk1/2 null LC appear to be more activated, which is known to trigger LC enlargement. Because Rsks are widely reported to promote cell growth through activation of protein translation via mTORC1 and other pathways (21), LC enlargement in Rsk1/2-null LC was unexpected. However, Rsks are not the sole activators of mTORC1 and, based on ribosomal S6 protein phosphorylation measured directly in LC, the activity of this key growth regulating kinase appeared unaffected by the absence of Rsks. Consistent with this, LC volume was essentially the same in Rsk1/2-null and WT adult epidermis. We suggest that increased LC surface area in Rsk1/2-null mice is achieved by a morphological change in response to reduced LC numbers. In the simplest case, this could be achieved by a flattening and broadening of the cells footprint without a substantial volume increase.

Several observations support the notion of an intrinsic inverse relationship between LC numbers and apparent LC size. First, LC surface area or footprint also correlated inversely with LC density in the epidermis of WT mice, indicating a normal behavioral feature of LC. Second, Rsk1/2-null bone marrow precursors were able to repopulate inflamed epidermis to the same density as WT precursors and showed normal size parameters. Third, upon exit from the epidermis, Rsk1/2-null LC assumed a normal size, again consistent with a tissue context-specific response. Fourth, a re-examination of the data in some earlier studies reveals an inverse correlation between LC size and density. Although this was sometimes due to LC activation triggering both increased LC size and LC exodus (45), in other cases, such as during post embryonic LC colonization of the epidermis (46) or repopulation after LC ablation (6), LC appear larger in underpopulated epidermis. Also, Kuipers et al. (47) reported that Dicer-deficient LC, although reduced in number, were ∼30% larger than WT LC, potentially reflecting a similar response. However, to our knowledge, our study is the first to explicitly document and measure this phenomenon and to investigate it in more detail.

Additional studies are needed to test the concept further, but an increase in LC footprint in response to reduced cell density would make biological sense because it could help offset potentially compromised surveillance of the epidermis due to reduced LC numbers. Although their dendrites appear largely sessile under resting conditions (6, 8), our results may also suggest that LC nonetheless sense the proximity of their neighbors and can respond accordingly.

## Supplementary Material

Data Supplement
