# Supplementary material for: The PDK1–Rsk Signaling Pathway Controls Langerhans Cell Proliferation and Patterning
Source: J Immunol. 2015 Sep 23;195(9):4264–72. doi: 10.4049/jimmunol.1501520 (PMC4640173; doi:10.4049/jimmunol.1501520)
Supplement: Data Supplement [file JI_1501520.zip › JI_1501520_Supplemental_Figures_1.pdf]

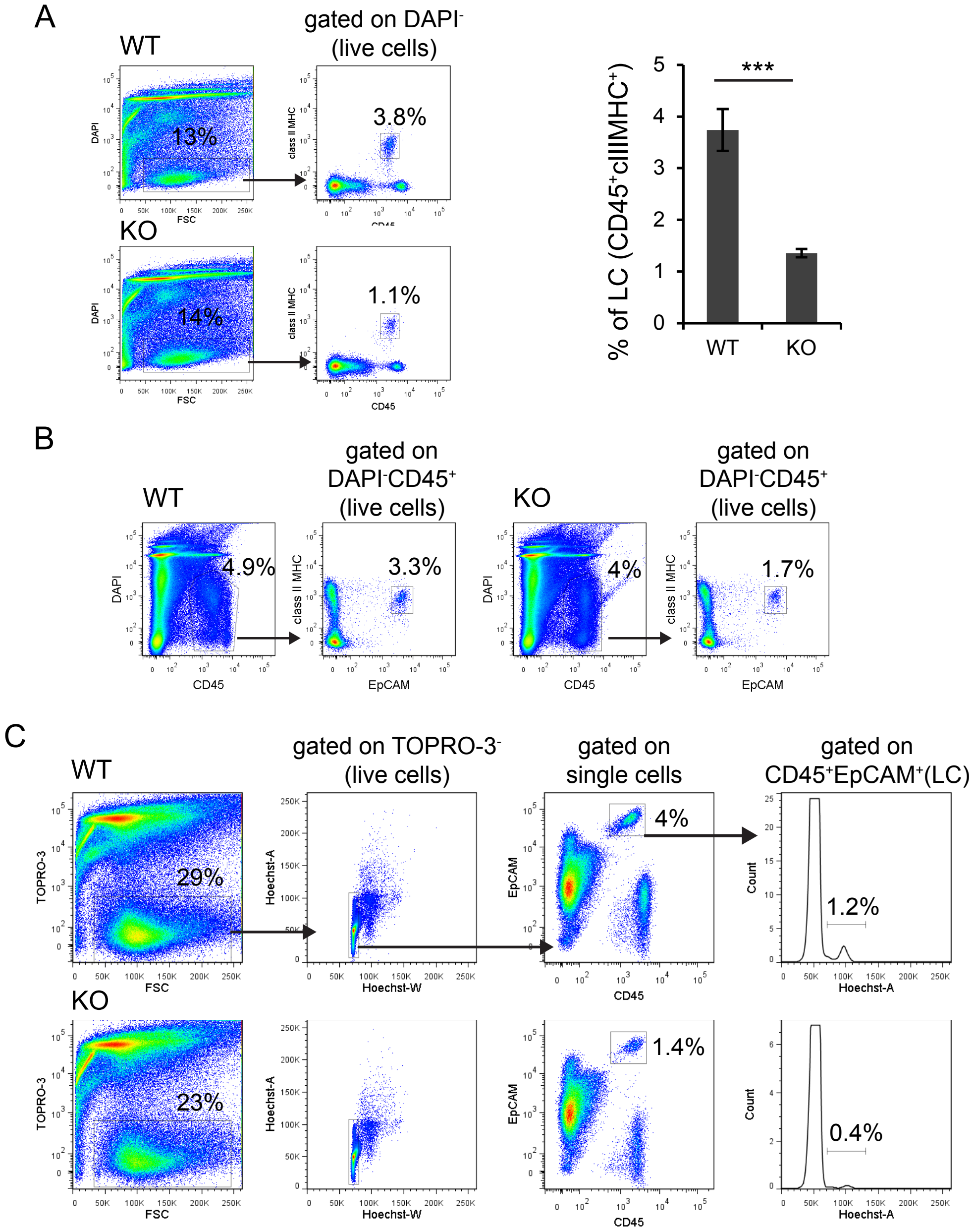

Supplemental Figure 1. (A-left) Representative dot plots showing the gating strategy to identify LC in either wt or Rsk1/2-null (KO) epidermis. The % of LC ($CD45^+$ class II MHC^+) is shown. (A-right) Histogram shows the % of LC in either wt or Rsk1/2-null epidermis. The data represent the mean $\pm$ SE (wt, n=6 and ko, n=7). (B) Representative dot plots showing the gating strategy to identify LC in wt or Rsk1/2-null dermis. The % of LC ($CD45^+$ $EpCAM^+$ class II MHC^+) is shown. (C) Representative dot plots showing the gating strategy to analyse LC cell cycle from either wt or Rsk1/2-null epidermis.

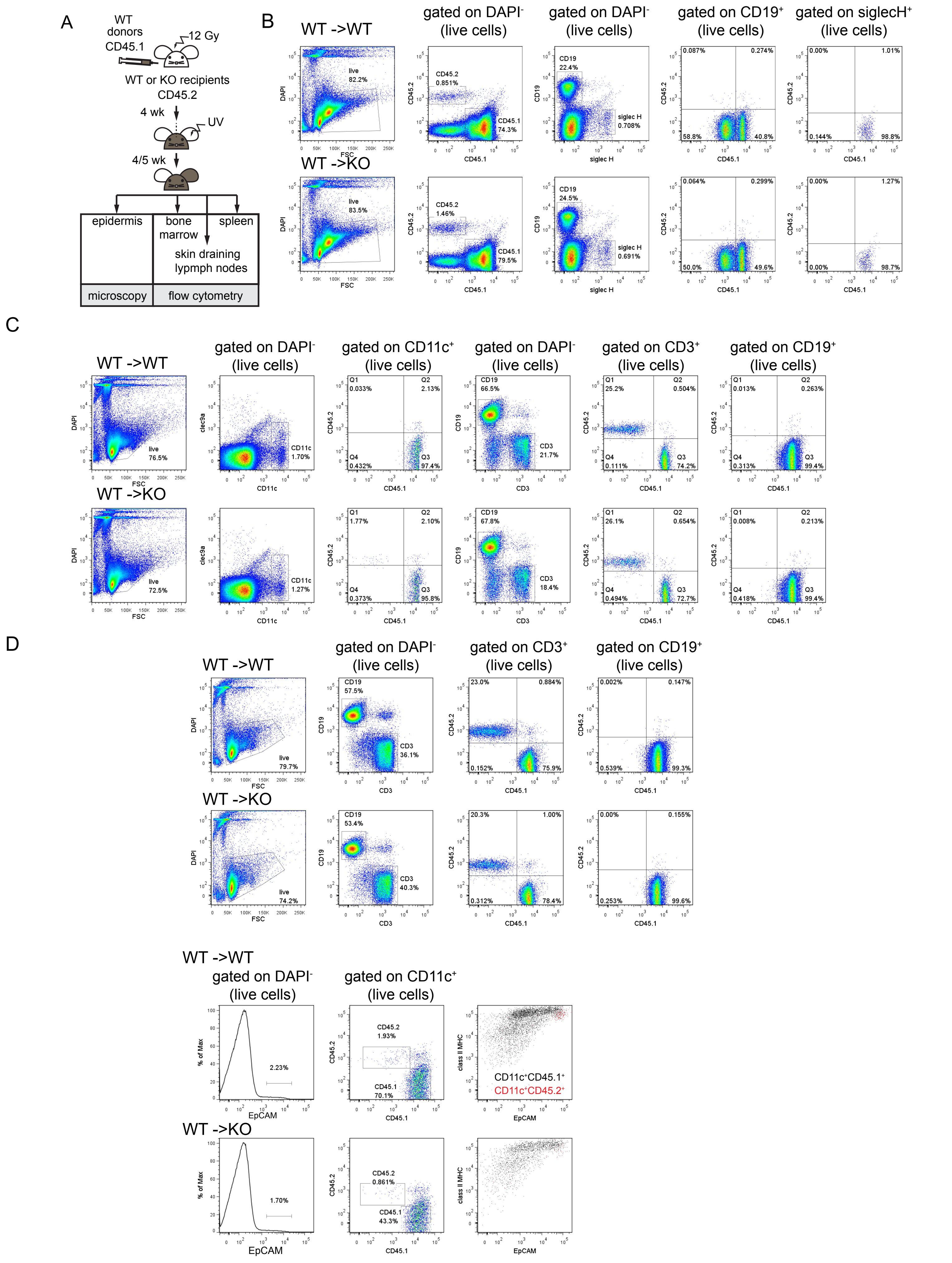

Supplemental Figure 2. Wt bone marrow reconstitutes wt and Rsk1/2-null irradiated mice with the same efficiency. (A) Experimental design. (B-D) Flow cytometry analysis of bone marrow (B), spleen (C) and skin draining lymph nodes (D) of wt or Rsk1/2-null (KO) mice reconstituted with wt bone marrow.

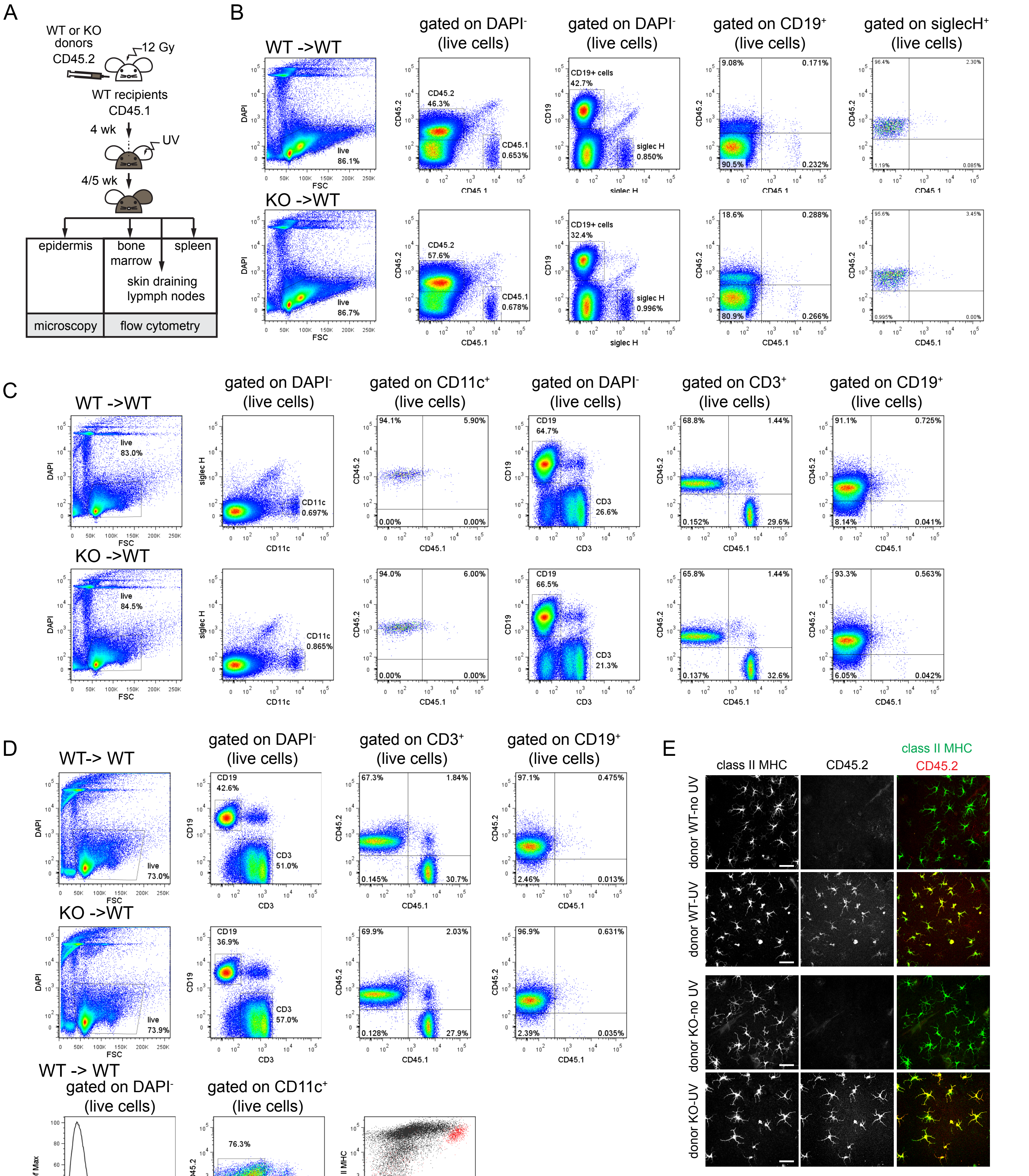

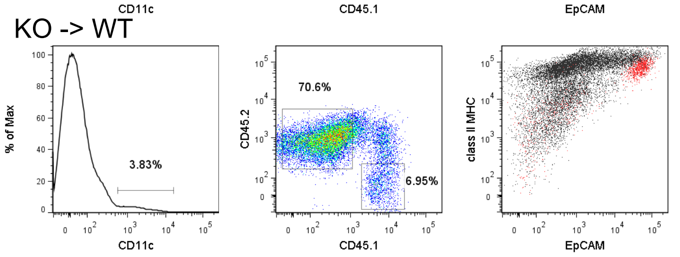

Supplemental Figure 3. Rsk1/2-null bone marrow reconstitutes wt irradiated mice with the same efficiency as wt bone marrow. (A) Experimental design. (B-D) Flow cytometry analysis of bone marrow (B), spleen (C) and skin draining lymph nodes (D) of wt mice reconstituted with either wt or Rsk1/2-null (KO) bone marrows. (E) Control (no UV) or UV treated epidermis was stained with FITC-labelled anti-class II MHC and APC-labelled CD45.2 Abs. Images were aquired on a LSM700 Zeiss confocal microscope as described. Bar 50 mm.
